# Step Count Accuracy of the Life Plus Connected Watch at Different Localizations and Speeds in Healthy Adults, Patients With Cardiovascular Disease, and Patients With Peripheral Artery Disease: Step Count Validation Study in Laboratory Settings

**DOI:** 10.2196/58964

**Published:** 2025-02-10

**Authors:** Anne-Noëlle Heizmann, Edouard Ollier, Pierre Labeix, Ivan Goujon, Frédéric Roche, Claire Le Hello

**Affiliations:** 1 INSERM, SAINBIOSE U1059, Saint-Priest-en-Jarez, France; 2 University Jean Monnet, Saint-Etienne, France; 3 Innovation and Pharmacology Clinical Research Unit, University Hospital of Saint-Etienne, Saint-Etienne, France; 4 Department of Physiology, University Hospital of Saint-Etienne, Saint-Etienne, France; 5 Department of Therapeutics and Vascular Medicine, University Hospital of Saint-Etienne, Saint-Etienne, France

**Keywords:** physical activity assessment, accuracy, validation, steps, smartwatches, wearables, cardiovascular disease, cardiovascular, cardiology, heart, artery, peripheral artery disease, wearable physical activity monitoring device, physical activity, exercise, walking, monitoring, mobile phone

## Abstract

**Background:**

Smartwatches are increasingly used to monitor and motivate physical activity. Patients with cardiovascular disease (CVD) and peripheral artery disease (PAD) often do not meet national physical activity recommendations. They may, thus, benefit from a physical activity program using smartwatches. The Life Plus smartwatch is designed to facilitate activity monitoring by counting steps, but its validity needs to be determined, particularly in patients who may not have a normal gait, such as those with cardiovascular pathology.

**Objective:**

This study evaluates the accuracy of the Life Plus smartwatch (versions 2 and 3) in healthy adults, patients with CVD, and patients with PAD at different walking speeds (1.8, 2.5, 3.2, and 4 km/h) and different localizations (wrists, hips, and ankles) to determine best accuracy.

**Methods:**

In total, 34 participants, comprising healthy individuals (n=10), patients with CVD (n=14), and patients with PAD (n=10), wore 6 Life Plus watches simultaneously (3 of version 2 and 3 of version 3), located on wrists, hips, and ankles. Participants walked on a treadmill for 3-minute sessions at speeds of 1.8, 2.5, 3.2, and 4 km/h; they then performed a 10-minute free walking on the ground and again walked for 3-minute sessions on a treadmill at the same speeds. Actual step counts were recorded through video footage.

**Results:**

When worn at the wrist, no significant difference between the actual number of steps and step count by version 2 watches was found in each group independently (healthy group: *P*=.25; CVD group: *P*=.50; and PAD group: *P*=.37)*.* Significant differences were found with the version 2 watches at the wrist in the healthy group at 3.2 (−5.26%; *P*=.01) and 4 km/h (−6.13%; *P*=.008) and in the CVD group at 2.5 (−5.94%; *P*=.008), 3.2(−13.1%; *P*=.008), and 4 km/h (−13.96%*; P*=.004). When worn at the wrist, no significant difference between actual number of steps and step count by version 3 watches was found in the healthy group (*P*=.02) and the PAD group (*P*=.90). Significant differences were found with the version 3 watches at the wrist in the CVD group at 3.2 (−6.43%; *P*=.001) and 4 km/h (−7.3%; *P*=.01) and in the PAD group at 4 km/h (−5.77%; *P*=.04).

**Conclusions:**

For optimized counting when worn at the wrist, healthy individuals should prefer version 2 watches for slow walking (1.8 and 2.5 km/h) and version 3 for higher speeds (3.2 and 4 km/h). Patients (CVD and PAD) should prefer version 2 watches at 1.8 km/h and version 3 watches for higher speeds (2.5, 3.2, and 4 km/h).

## Introduction

The benefits of regular physical activity on global health are well documented [1-4]. The key role of physical activity in cardiovascular diseases (CVDs) including peripheral artery disease (PAD) has been well reported within the scientific literature and has even been recognized as an effective nonmedical therapy and core component of cardiac rehabilitation [5,6]. Walking is the most natural physical activity that confers many benefits with minimal adverse effects [7-9]. In this context, daily step count seems to be an interesting target for achieving physical activity recommendations and setting physical activity goals [10]. Daily step count can easily be assessed and monitored by the patients themselves through wearable physical activity monitoring devices like a smartwatch. In healthy populations, wearable devices such as smartwatches are frequently used as motivational and self-monitoring tools for physical activity [11-14]. These wearable physical activity tracker devices offer considerable advantages in health care and personalized physical activity management in populations with chronic disease [15]. The gait of these patients can be different than those of healthy persons and may therefore influence the wearable device’s accuracy [16-19]. Patients with PAD are known to have an altered gait cycle and a reduction in step lengths [20-23].

The Life Plus connected watch is a wearable physical activity monitoring device that provides information about daily steps and daily walking activity. It also offers personalized and progressive goals, feedback, encouragements, and personalized innovative challenges, taking into account the users’ personal physical activity level and capacity. This watch was designed to be easy to use and not requiring a smartphone app, synchronization, or an internet connection. This watch seems to be an interesting tool to motivate patients to walk more. As this connected watch has not been evaluated, we wanted to estimate its accuracy in a healthy adult population as well as in populations with CVD or PAD. The aim of this study was to determine the accuracy of the Life Plus connected watch (versions 2 and 3) in counting steps under controlled treadmill walking conditions at different speeds in healthy adults and patients with CVD or PAD, with each participant wearing 6 watches at different locations (wrists, hips, and ankles). In view of the current literature, we hypothesize that the Life Plus watch is reliable for step counting with variations according to speeds, localizations, and the different groups (healthy adults, patients with CVD, and patients with PAD).

## Methods

### Recruitment

The inclusion criteria were adult healthy volunteers, adult persons with CVD or PAD (asymptomatic stage or stress ischemia stage) able to give written informed consent, to understand walking instructions to walk on a treadmill alone without technical help for several periods of 3 minutes at different speeds (1.8 to 4 km/h). Patients with CVD were recruited in the “Viellissement, Système Nerveux Autonome et Sommeil” (aging, autonomic nervous system, and sleep) center of the University Hospital of Saint-Etienne during their cardiac rehabilitation program. Patients with PAD were recruited in the Vascular Medicine Department of the University Hospital of Saint-Etienne during a scheduled ultrasound test for PAD. Investigators (CLH, ANH, FR, PL, and IG) explained the purpose and described the procedure to the patients who then decided whether or not they wanted to take part in the study. Healthy adults were volunteers recruited from medical and paramedical professionals at Saint-Etienne University Hospital, who gave their oral consent after a detailed presentation of the study. Exclusion criteria were volunteers and patients unable to walk independently, patients with PAD at chronic limb–threatening ischemia stage, and patients with walking limitations due to other pathologies. The ability to walk on a treadmill for 3 minutes at different paces was reported by all the patients recruited. None of the patients or healthy adults underwent any surgical procedures in the 3 months preceding the treadmill walking sessions.

### Ethical Considerations

This study was approved by the ethics committee of the University Hospital of Saint-Etienne in June 2022 (reference CE 2022‐36). The purpose of the study and the description of the procedure were explained to the participants before they signed an informed consent. Retrieved study data were deidentified and anonymized. No compensation was provided for participation.

### Material and Data Collection

Each participant wore simultaneously 6 Life Plus connected watches: 3 version 2 and 3 version 3, version 2 positioned on one side with one at the wrist, one at the hip, and one at the ankle; and version 3 on the other side in the same localizations. This disposition was determined to evaluate versions 2 and 3 Life Plus connected watches and to identify the best placement for better accuracy since the position may influence its accuracy [24]. Treadmill walking provided a controlled and easily replicable physical activity for evaluating the accuracy of step counting at different speeds by watches [25].

The Life Plus connected watch uses a triaxial accelerometer. Versions 2 and 3 are embedded with different accelerometers and step counting algorithms. Life Plus watches integrate a microelectromechanical system technology that is a spring-levered step counter. Step count recording was collected directly through the Life Plus watch screen. Videos of the participant’s lower limbs were recorded for a visual count of steps taken during all the walking sessions. Actual numbers of steps were counted a posteriori through visual inspection by the investigators with a camera of a Samsung Galaxy TAB S6 Lite under Android, which has a number of effective pixels of 12.8 megapixels (per million) with a full high-definition resolution of 1920×1080 (30 frames per screen).

The following data were retrieved: step counts by the watches, actual numbers of steps (videos), watch version (versions 2 and 3), group (healthy adults, patients with CVD, and patients with PAD), watch position (wrist, hip, and ankle), walking speed (1.8, 2.5, 3.2, and 4 km/h), help from the treadmill’s bars (yes and no), patients characteristics (age, BMI, and ankle-brachial index for patients with PAD).

### Study Design

**Figure 1.**
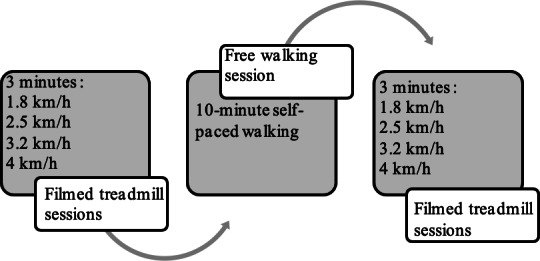
Study design.

After giving written informed consent, participants were invited to walk 4 sessions of 3 minutes on the treadmill at different speeds (1.8, 2.5, 3.2, and 4 km/h). As far as possible, participants were asked to let go of the treadmill bars as soon as they started their walking movement so that their arms could swing normally. Between each session, participants had to sit still on a chair for at least 2 minutes to allow step count data to be refreshed on the watch screen. After the first 4 sessions, the participants had a 10-minute free walking session at their own pace in the hospital corridor to simulate a fatigue pattern. Participants then again walked 4 sessions of 3 minutes on the treadmill at the same speeds as the first time. The treadmill walking sessions were filmed so that 2 investigators could count the actual number of steps taken during all the treadmill walking sessions (criterion measure). The study design is illustrated in Figure 1.

### Statistical Analysis

Each participant was assessed in 8 walking sessions at 1.8, 2.5, 3.2, and 4 km/h. In all the conditions, the difference between the step count recorded by the Life Plus connected watches and the actual number of steps counted by the investigators was computed. We calculated the quantiles Q1 and Q3 for the data on actual number of steps, then the IQR=Q3−Q1; then we calculated the lower and upper thresholds for the outliers with lower threshold=Q1−1.5*IQR and upper threshold=Q3+1.5*IQR. Outliers were removed from the data collected by the watches. For all analyses, the α significance threshold used was 5%. Concordance between the step count recorded by the connected watches and the actual number of steps was quantified by calculating the difference between the actual number of steps and the step count measured by the watches. These differences were analyzed using the Wilcoxon test as the Shapiro-Wilk test was performed and showed deviance from normality for actual number of steps and step count by watches.

ANOVA was computed to compare differences in age and BMI between groups, and the Tukey test was used to determine which differences were significant. Finally, Bland-Altman charts were constructed to evaluate the mean bias and the limits of agreement between step count by watches and actual numbers of steps. All analyses were conducted using the R Statistical language (version 4.0.4; R Foundation for Statistical Computing).

## Results

### Demographics

In total, 34 participants were included in the study: 10 in the healthy group, 14 in the CVD group, and 10 in the PAD group. Characteristics of the 3 groups are summarized in Table 1.

**Table 1.** Demographic characteristics of the 3 groups included in the Life Plus watch validation study (healthy adults, patients with cardiovascular disease [CVD], and patients with peripheral artery disease [PAD]).

	Healthy group (n=10)	CVD group (n=14)	PAD group (n=10)
Age (years), mean (SD)	32.5 (11)	59.7 (14.1)	65.1 (9.8)
**Sex, n (%)**			
Female	5 (50)	2 (14)	2 (20)
Male	5 (50)	12 (86)	8 (80)
BMI (kg/m^2^), mean (SD)	22.18 (2.98)	24.22 (1.89)	26.85 (4.03)
**Ankle-brachial index, mean (SD)**			
Right	—^a^	—	0.84 (0.28)
Left	—	—	0.88 (0.18)
**Toe pressure (mm Hg), mean (SD)**			
Right	—	—	78.6 (20.1)
Left	—	—	77.6 (26.6)

a Not available.

No significant age difference between the CVD and PAD groups (*P*=.53) was found, but significant differences between the healthy group and PAD group and the healthy group and CVD group (*P*<.001) were found. For BMI, analyses showed no difference between the CVD and PAD groups (*P*=.14) and between the healthy group and CVD group (*P*=.29) but significant differences between the healthy group and PAD group (*P*=.005).

### Evaluation Outcomes

Global statistical parameters are summarized in Table 2.

**Table 2.** Global statistical parameters for the number of steps counted by the investigators (number of actual steps) and counted by the Life Plus watches (versions 2 and 3).

	Number of actual steps	Number of steps measured by the watch(outliers removed)
Range	185-399	176-412
Median (IQR)	296.5 (264-324)	310 (277-343)
Mean (SD)	292.6 (43.73)	307.3 (49.71)

Independently from any condition, the Life Plus step watch overestimates the actual number of steps by mean+14.7 steps (*P*=.001; *r*=0.08; 95% CI 2-8.5).

### Life Plus Watch Version 2

Independently from all conditions, the version 2 watch did not correctly trigger automatic step counting (no step counted or less than the lower threshold of outliers) in 34% (n=81) walking sessions of the healthy group, 39% (n=116) in the CVD group, and 40% (n=95) in the PAD group. When looking closer, this technical issue occurred more frequently at slow speeds (1.8 and 2.5 km/h), when participants were holding the treadmill bars, and when the watch was worn at the hip or ankle. In the healthy group at 3.2 and 4 km/h when worn at the wrist, the version 2 watch triggered step counting in each walking session. In the CVD and PAD groups, at 3.2 and 4 km/h speeds, the version 2 watch triggered step counting more efficiently. Detailed statistics are summarized in Table S1 in Multimedia Appendix 1.

When worn at the wrist, no significant difference between actual number of steps and step count by watches was found (*P*=.89; 95% CI −8.0 to 9.0). The same result was found in each group independently (healthy group: *P*=.25; 95% CI −5.99 to −21; CVD group: *P*=.50; 95% CI −19.9 to 8.9; and PAD group: *P*=.37; 95% CI −8.0 to 22).

Detailed mean differences and mean percentage differences between the actual number of steps and step count by the watches are summarized in Table 3.

In the healthy group, when the watches were worn at the wrist, without holding treadmill bars and with speeds of 3.2 and 4 km/h, they underestimated the actual number of steps by mean 5.4% (*P*=.008 at 3.2 and 4 km/h). Differences between actual number of steps and step count by the watches were the lowest and nonsignificant when the watch was worn at the wrist at 1.8 km/h (*P*=.50; 95% CI 8-22) and 2.5 km/h (*P*=.21; 95% CI −22 to 3.5). In the CVD group, the mean percentage difference between the actual number of steps and the step count by the watches ranged from −13.96% (n=) at 4 km/h (*P*=.004; 95% CI −15.5 to −2.5) to −0.83% at 1.8 km/h (*P*=.13; 95% CI −126 to 24) when the watch was worn at the wrist. In the PAD group, the mean percentage difference between actual number of steps and the number of steps counted by the watches ranged from −12.44% at 4 km/h (*P*=.65; 95% CI −46 to 13.5) to +0.70% at 1.8 km/h (*P*=.81; 95% CI −49.5 to 35) when the watch was worn at the wrist.

**Table 3.** Mean step differences and mean percentage differences between actual number of steps and number of steps counted by version 2 watch in the 3 groups (healthy adults, patients with cardiovascular disease [CVD], and patients with peripheral artery disease [PAD]) at different localizations (wrist, hip, and ankle) and different speeds (1.8, 2.5, 3.2, and 4 km/h).

Speed and position		Groups		
		Healthy, mean step difference (%)	CVD, mean step difference (%)	PAD, mean step difference (%)
**1.8 km/h**				
	Wrist	9.37 (7.01)	−11.93 (−0.83)	−6.64 (0.70)
	Hip	17.94 (10.17)	−3.37 (2.33)	1.92 (3.86)
	Ankle	55.87 (23.85)	34.57 (16.02)	39.86 (17.54)
**2.5 km/h**				
	Wrist	2.24 (1.89)	−19.06 (−5.94)	−13.78 (−4.42)
	Hip	10.80 (5.06)	−10.50 (2.78)	5.21 (−1.26)
	Ankle	48.73 (18.74)	27.43 (10.90)	32.72 (12.43)
**3.2 km/h**				
	Wrist	−16.92 (−5.26)	−38.00 (−13.10)	−32.93 (−11.57)
	Hip	−8.36 (−2.10)	−29.66 (−9.94)	24.37 (−8.41)
	Ankle	29.58 (11.58)	8.28 (3.75)	13.56 (5.27)
**4 km/h**				
	Wrist	−18.76 (−6.13)	−40.06 (−13.96)	−34.78 (−12.44)
	Hip	10.20 (−2.96)	−31.50 (−10.80)	−26.21 (−9.27)
	Ankle	27.73 (10.72)	6.43 (2.88)	11.72 (4.41)

The Bland-Altman plots revealed no systematic differences between the actual number of steps and the number of steps counted by the watches for the 3 groups when the watch was worn at the wrist (Figure S1 in Multimedia Appendix 1).

### Life Plus Watch Version 3

Independently from all conditions, the version 3 watch did not correctly trigger automatic step counting (no step counted or less than the lower threshold of outliers) in 35% (n=83) of walking sessions in the healthy group, 43% (n=129) in the CVD group, and 43% (n=103) in the PAD group. When looking closer, this technical issue occurred more frequently at 1.8 and 2.5 km/h, when participants were holding the treadmill bars, and when the watch was worn at the hip or ankle. At 3.2 and 4 km/h and with the watch worn at the ankle, the watch version 3 triggered step counting more frequently. Detailed statistics are summarized in Table S2 in Multimedia Appendix 1.

When worn at the wrist, no significant difference between actual number of steps and number of steps counted by the watches (*P*=.52; 95% CI −15 to 7.0). The same result was found in the healthy group (*P*=.02; 95% CI 2.99-41.99) and the PAD group (*P*=.90; 95% CI −19 to +20). A significant difference was found in the CVD group (*P=.006; P*=.01; 95% CI −43 to +9) at higher speeds (3.2 and 4 km/h) but not at lower speeds (1.8 and 2.5 km/h). Mean differences and mean percentage differences between the actual number of steps and the number of steps counted by the watches are summarized in Table 4.

**Table 4.** Mean step differences and mean percentage differences between actual number of steps and number of steps counted by version 3 watch in the 3 groups (healthy adults, patients with cardiovascular disease [CVD], and patients with peripheral artery disease [PAD]) at different positions (wrist, hip, and ankle) and different speeds (1.8, 2.5, 3.2, and 4 km/h).

Speed and position		Groups		
		Healthy, mean step difference (%)	CVD, mean step difference (%)	PAD, mean step difference (%)
**1.8 km/h**				
	Wrist	28.28 (13.67)	6.98 (5.84)	12.27 (7.36)
	Hip	36.84 (16.83)	15.54 (8.99)	20.83 (10.52)
	Ankle	74.78 (30.51)	53.48 (22.68)	58.77 (24.20)
**2.5 km/h**				
	Wrist	21.15 (8.56)	−0.16 (0.72)	5.13 (2.25)
	Hip	29.71 (11.72)	8.41 (3.88)	13.70 (5.41)
	Ankle	67.64 (25.40)	46.34 (17.57)	51.63 (9.09)
**3.2 km/h**				
	Wrist	1.99 (1.40)	−14.03 (−6.43)	−19.31 (−4.91)
	Hip	10.55 (4.56)	−10.75 (−3.27)	−5.46 (−1.75)
	Ankle	48.49 (11.58)	27.18 (10.41)	32.47 (11.94)
**4 km/h**				
	Wrist	0.15 (0.54)	−21.16 (−7.30)	−15.87 (−5.77)
	Hip	8.71 (3.70)	−12.59 (−4.14)	−7.31 (−2.61)
	Ankle	46.64 (17.38)	25.34 (9.55)	30.63 (11.07)

Results showed that in the healthy group, differences between actual number of steps and steps counted by the watches were lower and nonsignificant when the watch was worn at the wrist at all speeds. In the healthy group, the version 3 watch tended to globally overestimate step counts in all conditions. In the CVD group, the mean percentage difference between actual number of steps and number of steps counted by the watch ranged from −7.29% (*P*=.01; 95% CI −32.5 to 108.5) at 4 km/h to +5.84% (*P*=.25; 95% CI −91 to 22) at 1.8 km/h when the watch was worn at the wrist. In the PAD group, the mean percentage of difference between the actual number of steps and the number of steps counted by the watches ranged from −5.77% (*P*=.04; 95% CI −59 to −4) at 4 km/h to +7.36% (*P*=1.0; 95% CI −156 to 81) at 1.8 km/h when the watch was worn at the wrist.

The Bland-Altman plots revealed no systematic differences between the actual number of steps and the step count obtained by the watches when the watch was worn at the wrist (Figure S2 in Multimedia Appendix 1).

## Discussion

### Principal Findings

Activity trackers were designed to help people to monitor their daily physical activity. A smartwatch able to accurately count the number of steps and provide motivating features, such as the Life Plus watch, would benefit patients’ physical activity. Indeed, it is important to have a reliable activity tracker [26] to support and coach patients in home-based physical activity rehabilitation. Inaccurate information on physical activity may lead to frustration for the patient if his activity is underestimated or to false positive feedback if overestimated. As these tools often are tested in healthy populations, we wanted to test the accuracy of the Life Plus watch for counting steps in healthy volunteers, patients with CVD, and patients with PAD.

On the basis of our investigation, the Life Plus smartwatches seemed to be accurate for counting steps in healthy persons when walking on a treadmill without holding the lateral bars. The version 3 version was better in counting steps.

Our results indicated that in the healthy group, when worn at the wrist, the version 2 watches were more accurate at 1.8 and 2.5 km/h; whereas at 3.2 and 4 km/h, the version 3 watches were more accurate. Healthy adults should wear the watch, version 2 or 3, at the wrist for better accuracy. In the CVD and PAD groups, versions 2 and 3 watches have similar accuracy at 1.8 and 2.5 km/h when worn at the wrist or hips; at 3.2 and 4 km/h, the version 3 watches are more accurate than the version 2 when worn at the wrist or hips. In practice, the version 3 watch seems to be the better option in all groups with better accuracy at all speeds when worn at the wrist or hips.

Differences between the number of steps counted by watches according to their version (versions 2 and 3) are explained by the different accelerometers and corresponding step counting algorithm embedded in versions 2 and 3.

### Comparison With Prior Work and Limitations

Globally, our investigation showed that step count accuracy decreased as walking speed decreased, which is consistent with other validation studies [27,28]. In our study design, speeds were not counterbalanced; therefore, we could not ensure that there was no order effect on step counts.

The localization of the watch on the body also influences the step count accuracy, with overestimation when worn at the ankles, and underestimation when worn at the hips at higher speeds. This result follows other validation studies [29].

The watch had slightly lower accuracy in the CVD and PAD groups. This could be explained by the fact that the patients were more often holding the treadmill lateral bars to maintain stability, and wrist-worn wearable devices typically undercount the number of steps when hands are fixed on lateral hand bars [30]. Moreover, patients may have had some impaired arm swing and gait pattern as shown in several studies [17,18,20-23]. Furthermore, age, sex, and BMI also may influence the validity of wearable devices [31]. These could also have affected our results as the mean age of the healthy participant’s group was much lower than that of the 2 disease groups. This makes the comparison between groups difficult. Results could also be influenced by the fact that the study design did not propose a familiarization session on the treadmill. Walking on a treadmill is not natural and requires a lot of attention at first. Several studies showed indeed higher cadences, shorter step lengths, shorter swing phases, longer stance phases, and higher walking variability when walking on a treadmill [32,33]. A 10-minute session to familiarize with walking on a treadmill could enable to be in conditions nearer to natural walking [34].

Participants only walked for 3 minutes for each speed. The accelerometer has a wake-up threshold, meaning it only starts measuring movement when a certain level of acceleration is detected. At slower speeds, the arm swing may not be sufficiently pronounced to reach the accelerometer’s step count threshold. The algorithm has set specific thresholds that need to be met for a movement to be recognized as a step. If the arm swing is too subtle, it may not reach these thresholds, resulting in steps not being counted. This could explain the percentage of errors in step count.

Our research evaluated the accuracy of the Life Plus watch, versions 2 and 3, in laboratory conditions. Future research should investigate the accuracy of the watch during free-living conditions.

### Conclusions

We investigated the accuracy of versions 2 and 3 of the Life Plus watch in healthy adults, patients with CVD, and patients with PAD. Accuracy of the version 3 was better for all walking speeds when worn at the wrist. Mean percentage differences between actual number of steps (videos) and number of steps counted by watches were less than 10% when version 3 was worn at the wrist by each group at all speeds except 1.8 km/h. Globally, independently from the watch version, accuracy slightly decreased when worn by patients with CVD or PAD walking at slower speeds. In the CVD and PAD groups, the accuracy of version 3 was better when worn at the wrist for slower walking speeds (1.8 and 2.5 km/h) and when worn at the hips for higher walking speeds. In practice, when worn at the wrist as it is intended, healthy individuals should prefer version 2 watches for slow walking (1.8 and 2.5 km/h) and version 3 for higher speeds (3.2 and 4 km/h). Patients (CVD and PAD) should prefer version 2 watches at 1.8 km/h and version 3 watches for higher speeds (2.5, 3.2, and 4 km/h).

## Supplementary material

10.2196/58964 Multimedia Appendix 1 Outliers table and Bland-Altman plots.

## Abbreviations

CVD: cardiovascular disease
PAD: peripheral artery disease
