# Supplementary material for: Step Count Accuracy of the Life Plus Connected Watch at Different Localizations and Speeds in Healthy Adults, Patients With Cardiovascular Disease, and Patients With Peripheral Artery Disease: Step Count Validation Study in Laboratory Settings
Source: JMIR Form Res. 2025 Feb 10;9:e58964. doi: 10.2196/58964 (PMC11833183; doi:10.2196/58964)
Supplement: Multimedia Appendix 1 [file formative-v9-e58964-s001.docx]

**Table S1.** Percentage of outliers in each group, position and speed for the V2 Life Plus watch.

| **Speed** | **Position** | **groups** | | |
| --- | --- | --- | --- | --- |
|  |  | Healthy group | CVD group | PAD group |
|  |  |  |  |  |
| **1.8 km/h** | Wrist | 90 | 84 | 65 |
|  | Hips | 85 | 88 | 50 |
|  | Ankle | 25 | 44 | 50 |
| **2.5 km/h** | Wrist | 35 | 40 | 45 |
|  | Hips | 40 | 24 | 50 |
|  | Ankle | 25 | 32 | 30 |
| **3.2 km/h** | Wrist | 0 | 24 | 45 |
|  | Hips | 15 | 12 | 25 |
|  | Ankle | 40 | 32 | 35 |
| **4 km/h** | Wrist | 0 | 24 | 35 |
|  | Hips | 10 | 12 | 10 |
|  | Ankle | 40 | 48 | 35 |

Abbreviations: CVD, cardiovascular disease; PAD, peripheral artery disease.

**Table S2.** Percentage of outliers in each group, position and speed for the V3 Life Plus watch.

| **Speed** | **Position** | **Groups** | | |
| --- | --- | --- | --- | --- |
|  |  | Healthy group | CVD group | PAD group |
|  |  |  |  |  |
| **1.8 km/h** | Wrist | 65 | 84 | 80 |
|  | Hips | 80 | 92 | 70 |
|  | Ankle | 10 | 28 | 40 |
| **2.5 km/h** | Wrist | 45 | 68 | 65 |
|  | Hips | 80 | 60 | 25 |
|  | Ankle | 10 | 12 | 25 |
| **3.2 km/h** | Wrist | 30 | 28 | 60 |
|  | Hips | 40 | 48 | 20 |
|  | Ankle | 10 | 4 | 30 |
| **4 km/h** | Wrist | 15 | 52 | 55 |
|  | Hips | 25 | 36 | 10 |
|  | Ankle | 5 | 4 | 35 |

Abbreviations: CVD, cardiovascular disease; PAD, peripheral artery disease.

**Figure S1.** Bland Altman Plots for V2 watches when worn at the wrist.

**Figure S2**. Bland Altman Plots for V3 watches when worn at the wrist.
